# Quinine Water-Triggered Atrial Tachyarrhythmia

**DOI:** 10.7759/cureus.32706

**Published:** 2022-12-19

**Authors:** Emad Elmusa, Hannan Asghar, Ameer Hamza, Muhammad Waleed Raza, Ismael Rodriguez

**Affiliations:** 1 Internal Medicine, HCA Florida Orange Park Hospital, Orange Park, USA

**Keywords:** atrial tachyarrhythmia, tachyarrhythmia, atrial flutter, atrial flutter with rapid ventricular response, tonic water, quinine

## Abstract

Quinine is an anti-malarial drug with documented hematologic, dermatologic, and cardiovascular side effects. Tonic water contains a sub-therapeutic amount of quinine and is available over the counter. However, the public is unaware of the risks associated with excessive consumption of tonic water. We present a patient who developed atrial flutter with a rapid ventricular response following the consumption of tonic water. The patient responded to rate control therapy and was discharged the following day with a plan to follow up in the outpatient department with an electrophysiologist. Although quinine has been shown to have ventricular anti-arrhythmic effects, its effect on the atria has not been determined. We present this case to bring greater awareness to the cardiovascular risks associated with the consumption of tonic water to reduce morbidity and mortality.

## Introduction

Quinine is an anti-malarial drug and its mechanism of action is unknown, but it is thought to inhibit nucleic acid and protein synthesis. Quinine is also able to block cardiac myocyte sodium channels and has been shown to suppress ventricular arrhythmias [1]. However, its effect on the atrial myocardium has not been determined [1]. Atrial fibrillation and flutter are common atrial arrhythmias. There are multiple known etiologies and triggers of these arrhythmias. They are associated with significant morbidity and mortality when there is a rapid ventricular rate. We present a patient who developed atrial flutter with a rapid ventricular rate following ingestion of tonic water. A workup revealed that the patient’s arrhythmia was most likely secondary to his tonic water ingestion because of its quinine content.

## Case presentation

A 75-year-old Caucasian male presented to the emergency department with a chief complaint of palpitations and a fast heart rate. The patient denied any similar episodes in the past. Upon further evaluation, he endorsed consuming 1 liter of tonic water prior to the onset of his symptoms-his first time drinking tonic water in the last five years.

The patient’s past medical history was significant for hypertension, hyperlipidemia, and prostate cancer in remission following treatment with proton therapy. Cardiac history one year prior to presentation was significant for event monitoring for electrocardiographic premature ventricular contractions, which revealed normal sinus rhythm, a negative stress test, and an unremarkable echocardiogram with preserved ejection fraction.

**Figure 1:**
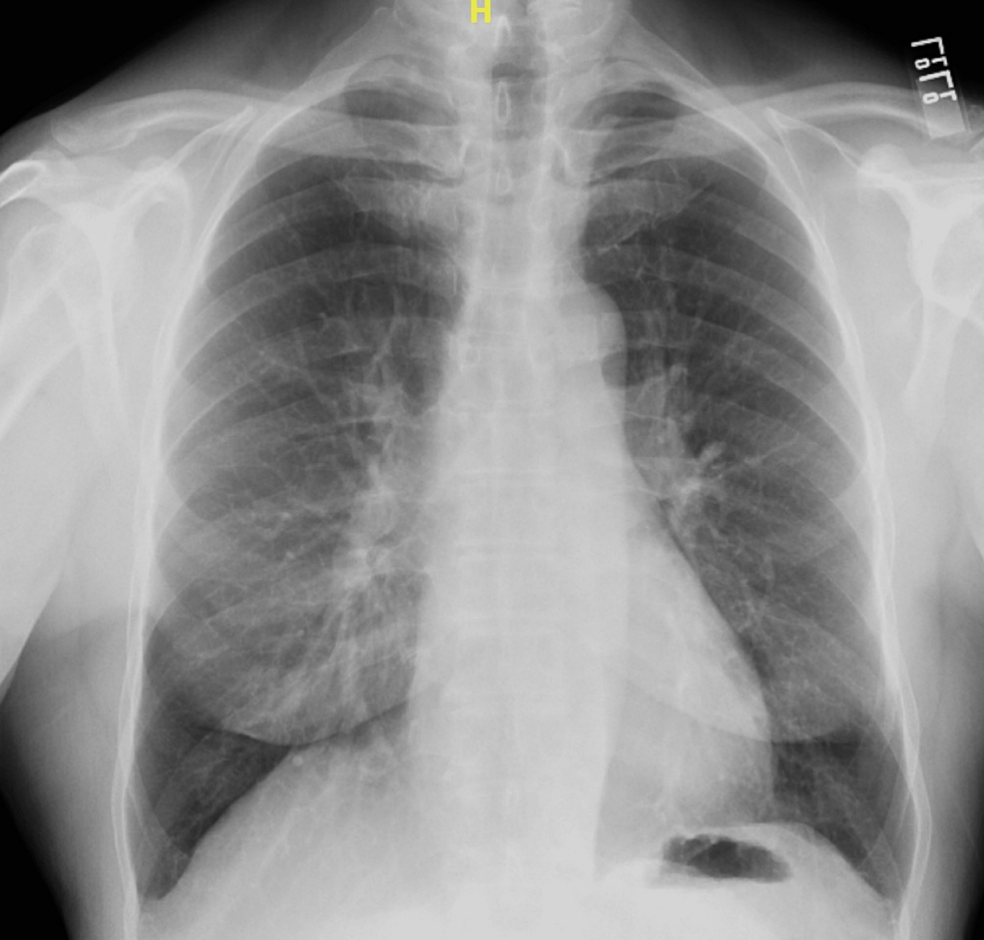
Normal appearing chest X-ray

On admission, our patient denied chest pain or lightheadedness. Vitals revealed a heart rate of 120-140 beats per minute (bpm), blood pressure of 147/94 mmHg, and pulse oxygen saturation of 97%. Physical examination was only significant for a rapid pulse which was irregular. Biochemical investigations were significant for creatinine of 1.52 mg/dL (reference 0.55 - 1.02 mg/dL). High-sensitivity cardiac troponin, electrolytes, urinalysis, and thyroid-stimulating hormone were normal. Chest X-ray was normal, it revealed no acute cardiopulmonary disease process (Figure 1). 

**Figure 2:**
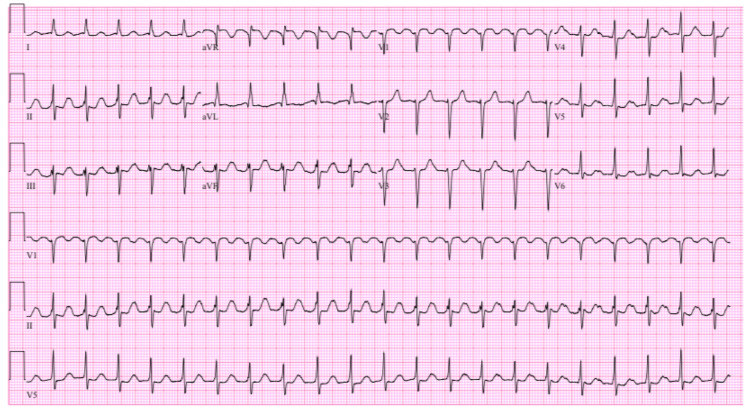
Atrial flutter with 2:1 AV conduction and a heart rate of 128 beats per minute AV: atrioventricular

**Figure 3:**
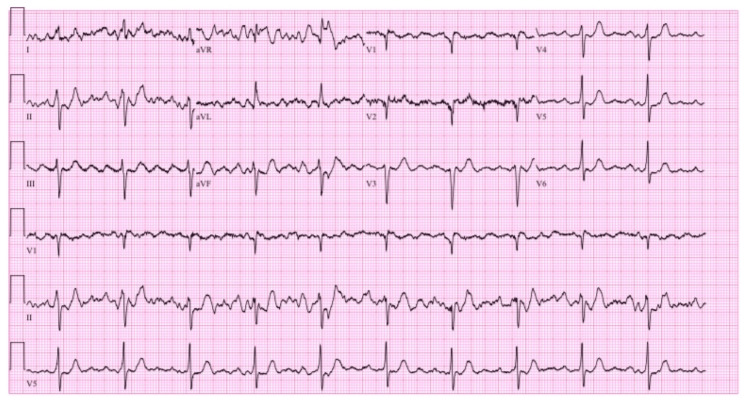
Atrial flutter with 4:1 AV conduction and a heart rate of 63 beats per minute AV: atrioventricular

An initial electrocardiogram (EKG) revealed atrial flutter with a 2:1 atrioventricular (AV) conduction and a heart rate of 128 beats per minute (Figure 2). The patient was given one 15 mg/kg intravenous (IV) bolus of diltiazem followed by a diltiazem titratable infusion. Repeat EKG after the bolus of diltiazem revealed atrial flutter with 4:1 AV conduction and a heart rate of 63 beats per minute (Figure 3), a significant improvement from the prior EKG. The patient's calculated CHA₂DS₂-VASc score was 3, two points for age and one point for hypertension. The patient was given a 70 units/kg bolus of heparin and started on a titratable heparin infusion to reduce his risk of stroke.

On the following day, the creatinine improved to 1.05 mg/dL after infusion of one liter of isotonic fluids. The patient's calculated fractional excretion of sodium was 7%. This indicates post-renal injury, likely post-obstructive relating to his history of prostate cancer.

The diltiazem infusion was weaned off and the patient’s home oral metoprolol succinate 25 mg daily was resumed, with additional metoprolol tartrate IV as needed to maintain a heart rate below 110 beats per minute. The patient maintained a heart rate between 60-80 beats per minute. We transitioned the IV heparin to oral rivaroxaban and increased metoprolol succinate to 50 mg daily, with directions to follow up in the outpatient department with an electrophysiologist. The patient was also educated on avoiding any additional consumption of tonic water. 

## Discussion

Quinine is used to treat malaria and babesiosis. In the United States, quinine and related compounds are not available for over-the-counter purchases. However, water containing quinine is commonly available for over-the-counter purchase. This is problematic because of quinine’s adverse side effect profile. Quinine is associated with cinchonism, hemolytic uremic syndrome, disseminated intravascular coagulation, leukopenia, and neutropenia [1-6]. Additionally, quinine is associated with cardiovascular side effects-including QRS, and QT prolongation [7]. Quinine is a class 1 anti-arrhythmic that binds and inhibits cardiac myocyte sodium channels [7].

We described a case of quinine-induced atrial tachyarrhythmia in a 75-year-old male following recreational consumption of tonic water. Quinine has documented anti-arrhythmic effects within the ventricular myocardium-effective in both ventricular fibrillation and premature ventricular contractions [7]. Quinine’s effect on the atrial myocardium is not well-established [7]. However, just as quinine binds and inhibits sodium channels within the ventricular myocardium, it is also likely that it is able to bind and inhibit sodium channels within the atrial myocardium. It is possible that our patient had ectopic atrial electrical foci prior to the ingestion of tonic water. After ingestion, the quinine likely inhibited depolarization of the patient’s atrial myocardium and subsequently resulted in electrical impulses through ectopic atrial foci. This is evidenced by the persistence of atrial flutter after atrioventricular node blockade therapy.

The FDA limits tonic water quinine content to 83 mg/L and therapeutic quinine doses typically range between 500 to 1000 mg [8]. Although tonic water contains sub-therapeutic levels of quinine and although atrial flutter is common-especially in the elderly, it appears unlikely that the observed association was coincidental. We propose there is still a risk as demonstrated in this case. Other causes that trigger atrial flutter-such as structural heart, pulmonary, and endocrine abnormalities-were excluded.

## Conclusions

This case highlights the importance of considering over-the-counter products, including tonic water, in the differential diagnosis for triggers of atrial arrhythmias. Atrial arrhythmias are associated with significant morbidity and mortality when there is a rapid ventricular rate. Therefore, warning labels on tonic water may be needed to prevent cardiovascular morbidity and mortality in at-risk individuals. We propose avoiding excessive tonic water due to its unpredictable effects on the cardiovascular system.
